# Molecular Classification as a Predictor of Nodal Involvement and Survival Outcomes in Presumed Early-Stage Endometrial Cancer

**DOI:** 10.3390/cancers18101628

**Published:** 2026-05-18

**Authors:** Irene Pellicer, Blanca Diaz, María Espías-Alonso, Ignacio Zapardiel, Myriam Gracia

**Affiliations:** Gynecologic Oncology Department, La Paz University Hospital, 28046 Madrid, Spain; i.pellicerespinosa@gmail.com (I.P.); blancadifuentes@gmail.com (B.D.); ignaciozapardiel@hotmail.com (I.Z.); dra_gracia@hotmail.com (M.G.)

**Keywords:** endometrial cancer, molecular classification, POLE, MMR, p53, sentinel lymph node, survival, prognostic factors

## Abstract

**Background:** Molecular classification has transformed risk stratification in endometrial cancer, providing prognostic information beyond traditional clinicopathologic features. However, the relationship between molecular subtype, nodal involvement, and recurrence risk remains incompletely defined. This study aimed to compare lymph node metastasis rates across molecular subgroups and evaluate survival outcomes and prognostic factors for recurrence. **Methods:** We conducted a retrospective study including 158 patients with a preoperative diagnosis of presumed early-stage endometrial carcinoma treated surgically between 2021 and 2024. Molecular classification was performed according to WHO criteria, including POLE-ultramutated, mismatch repair deficient (MMRd), p53-abnormal (p53-abn), and no specific molecular profile (NSMP). Sentinel lymph node biopsy (SLNB) was the primary method for nodal staging. Survival outcomes were assessed using a Kaplan–Meier analysis, and logistic regression was used to identify prognostic factors for recurrence. **Results:** NSMP was the most frequent molecular subtype (44.3%), followed by MMRd (29.1%), p53-abn (20.9%), and POLE-mutated tumors (5.7%). Overall, 11.4% of patients had nodal metastases, most commonly in the p53-abn subgroup, which showed significantly higher rates of positive sentinel lymph nodes (*p* = 0.010). Prognosis differed significantly across molecular subtypes. POLE-mutated and NSMP tumors demonstrated the most favorable outcomes, while p53-abn tumors showed the poorest overall survival and progression-free survival. In a univariate analysis, grade, lymphovascular space invasion (LVSI), myometrial invasion, FIGO stage, and molecular classification were associated with recurrence. Stratified analyses suggested LVSI as the most relevant prognostic factor within the MMRd subgroup. **Conclusions:** Molecular classification is strongly associated with nodal involvement and survival outcomes in early-stage endometrial cancer. Integrating molecular subtype with clinicopathologic factors may improve recurrence risk stratification and guide individualized surgical and adjuvant treatment strategies.

## Simple Summary

Molecular classification has significantly improved risk stratification in endometrial cancer, complementing traditional clinicopathologic parameters. This retrospective study included 158 patients with a preoperative diagnosis of presumed early-stage disease treated surgically between 2021 and 2024, aiming to evaluate the association between molecular subtype, lymph node metastasis, and recurrence risk. Tumors were classified according to WHO criteria into POLE-mutated, MMR-deficient (MMRd), p53-abnormal (p53-abn), and no specific molecular profile (NSMP). Sentinel lymph node biopsy was the primary nodal staging method. NSMP was the most frequent subtype (44.3%), followed by MMRd (29.1%), p53-abn (20.9%), and POLE-mutated tumors (5.7%). Overall, 11.4% of patients presented nodal metastases, with significantly higher rates observed in the p53-abn subgroup. Survival outcomes differed across molecular subtypes, with POLE-mutated and NSMP tumors showing the most favorable prognosis, while p53-abn tumors demonstrated the poorest survival. Lymphovascular space invasion (LVSI) emerged as the most relevant prognostic factor for recurrence within the MMRd subgroup. Integrating molecular classification with clinicopathologic factors may improve individualized risk assessment and treatment strategies.

## 1. Introduction

Endometrial carcinoma is the most common gynecologic cancer in developed countries, with an increasing incidence due to population aging and rising obesity rates [1,2]. In the United States alone, epidemiological models project more than 69,000 new cases per year [3]. Globally, the incidence continues to rise due to the growing prevalence of risk factors, such as obesity, diabetes, and hypertension [1,2,3,4].

The molecular classification introduced by The Cancer Genome Atlas (TCGA) in 2013 has transformed the understanding and management of endometrial carcinoma by defining four biological subtypes: POLE-ultramutated, mismatch repair-deficient (MMRd), p53-abnormal (p53-abn), and no specific molecular profile (NSMP) [5,6]. This classification has been incorporated into the World Health Organization (WHO) recommendations and international guidelines such as the European Society of Gynaecological Oncology (ESGO). Integrating immunohistochemistry for MMR proteins and p53 with sequencing for pathogenic POLE mutations enhances prognostic stratification beyond traditional histopathologic evaluation [1,4,5,6].

Recent refinements of molecular classification have further enhanced its clinical relevance. In 2025, estrogen receptor (ER) status emerged as a key prognostic discriminator within the NSMP subgroup [7,8]. The current guidelines recommend routine ER and progesterone receptor (PR) assessment in advanced and recurrent disease. ER-positive NSMP tumors (cutoff > 10%) are associated with favorable outcomes and low disease-specific mortality, whereas ER-negative NSMP tumors exhibit aggressive clinical behavior [9,10].

Each molecular subtype is associated with distinct clinical outcomes: POLE-ultramutated tumors have an excellent prognosis. MMRd and ER-positive NSMP tumors show intermediate outcomes, while p53-abn and ER-negative NSMP tumors carry the worst prognosis [1,4,7,8,9]. Beyond prognostic stratification, molecular classification also predicts response to therapy, including radiotherapy, chemotherapy, and immunotherapy. Immunotherapy has transformed the management of advanced and recurrent disease, particularly in MMRd and MSI-H subtypes. Furthermore, ER expression independently predicts improved disease-free survival (DFS) and overall survival (OS) and supports the use of hormonal therapy for ER-positive NSMP tumors and treatment intensification strategies for ER-negative NSMP tumors, akin to p53-abn disease [1,4,7,8,9].

In the same years, surgical staging has evolved with the widespread adoption of sentinel lymph node (SLNB) as the preferred method for nodal assessment in clinically uterine-confined endometrial cancer. Prospective trials, including the landmark FIRES trial, have demonstrated high sensitivity and a negative predictive value for detecting nodal metastases, even in high-risk histologies with lower morbidity than systematic lymphadenectomy [1,10,11]. Consequently, SLNB is now endorsed by both the National Comprehensive Cancer Network (NCCN) and European guidelines as the standard approach for nodal staging in apparent early-stage disease [1,2,10].

The integration of molecular profiling with sentinel lymph node (SLN) mapping has the potential to revolutionize endometrial cancer management. However, the prognostic significance of molecular classification in patients with nodal metastases remains incompletely defined. Emerging studies regarding this suggest that p53-abn tumors exhibit the highest rates of nodal involvement, followed by MMRd, NSMP, and POLE-ultramutated tumors [12,13,14,15]. Despite the prognostic impact of molecular subtype, conventional histopathologic parameters such as tumor grade or extent of nodal involvement continue to play critical roles. These findings highlight the necessity of integrating risk assessment models.

In this context, the present study aims to compare the rates of nodal involvement across molecular subgroups and to evaluate survival according to molecular classification. As a secondary objective, we sought to investigate clinicopathologic prognostic factors for recurrence within the different molecular subgroups.

## 2. Materials and Methods

### 2.1. Study Design and Setting

A retrospective study including all consecutive patients with histologically confirmed endometrial carcinoma treated surgically at La Paz University Hospital between January 2021 and December 2024 was conducted. The study was conducted in accordance with the Declaration of Helsinki and local ethical requirements.

Eligible patients included adult women (≥18 years) with a preoperative diagnosis of early-stage endometrial carcinoma who underwent complete surgical staging. Pathologic confirmation was required by endometrial biopsy. Patients were excluded if they were diagnosed with a synchronous invasive malignancy at the moment of surgical treatment or with advanced endometrial disease. Other exclusions were neoadjuvant therapy received before definitive surgery, inadequate tissue for molecular testing, or if the patient was unavailable for follow-up after surgery.

### 2.2. Preoperative Assessment and Surgical Management

Preoperative diagnosis of endometrial carcinoma was histologically confirmed by hysteroscopy biopsy in all cases. According to the ESGO guideline [2], preoperative assessment included ultrasound or magnetic resonance imaging (MRI) in low or intermediate-risk endometrial carcinoma with low-grade histology. Excluding these cases, a computed tomography (CT) scan or positron emission tomography (PET-CT) scan was performed to rule out local or distant metastasis.

All patients were newly diagnosed for presumed early-stage endometrial carcinoma and underwent complete surgical staging by total hysterectomy with bilateral adnexectomy and lymph node assessment (SLNB and/or systematic pelvic with or without para-aortic lymphadenectomy). Minimally invasive surgery (MIS), conventional laparoscopy, or robotic surgery was the preferred surgical approach. However, in some cases, such as big uterine fibroids, the selected surgical approach was laparotomy. Intraoperatively, the detection of SLN was performed by using indocyanine green tracer (ICG).

### 2.3. Molecular Classification

Molecular analysis was routinely performed on the preoperative endometrial biopsy and completed or extended after the final histopathological study of the surgical specimen. Molecular classification was performed according to the WHO Classification of Female Genital Tumours (5th/6th edition), incorporating an immunohistochemical assessment of MMR proteins and p53, together with POLE sequencing when indicated [16].

Tumors were categorized into four molecular subgroups according to current WHO and ESGO recommendations: POLE-ultramutated (pathogenic exonuclease domain mutation), MMRd, p53-abn, and NSMP. Immunohistochemistry for MMR proteins and p53 was routinely performed in all cases. POLE sequencing was performed for all tumors except for low-grade endometrioid carcinomas that were MMR-proficient and p53 wild type, in which POLE testing was not carried out according to our institutional algorithm. ER and PR expression were assessed by immunohistochemistry.

Postoperative ultrastaging of SLN was performed by multiple sectioning at 200 μm intervals or one-step nucleic acid amplification [17]. Lymph node metastases were classified as isolated tumor cells (ITCs, ≤0.2 mm), micrometastasis (0.2–2 mm), and macrometastasis (>2 mm). Only in one case were ITCs identified in the cohort. This case was not considered as nodal positivity for the purposes of postoperative staging, in accordance with current staging criteria, and, therefore, was not classified as a node-positive disease in the primary analysis. All pathologic diagnoses were confirmed by an expert gynecologic pathologist. However, a centralized, blinded pathology review was not performed, and cases were not systematically double-read or evaluated in a formal consensus setting. Histopathological assessment, including evaluation of LVSI, was therefore based on routine clinical practice, which may be subject to interobserver variability.

### 2.4. Data Collection

Clinical, surgical, pathological, and follow-up data were extracted from electronic medical records. Variables included age, body mass index (BMI), International Federation of Gynaecology and Obstetrics (FIGO 2023) stage, histologic subtype, tumor grade, LVSI, myometrial invasion, nodal status, molecular subtype, adjuvant treatment, recurrence, and survival outcomes. DFS was defined as the time from surgery to recurrence or last follow-up. OS was defined as the time from surgery to death from any cause or last follow-up.

For regression analyses, clinicopathological variables were coded as follows: histology was classified as endometrioid versus non-endometrioid; tumor grade as low grade (G1–G2) versus high grade (G3); myometrial invasion as <50% versus ≥50%; LVSI as positive versus negative; and lymph node involvement as positive versus negative. Prognostic risk classification was defined according to the ESGO criteria as low risk, intermediate risk, intermediate–high risk, high risk, or indeterminate risk.

### 2.5. Statistical Analysis

Continuous variables are presented as median and interquartile range (IQR) or mean ± standard deviation (SD), and categorical variables as frequencies and percentages. Comparisons between molecular groups were performed using chi-square or Fisher’s exact test for categorical variables and ANOVA or Kruskal–Wallis test for continuous variables, as appropriate. Survival curves were estimated using the Kaplan–Meier method and compared using the log-rank test. Univariate and multivariate logistic regression analyses were conducted to identify prognostic factors for recurrence. Odds ratios (ORs) with 95% confidence intervals (CIs) were calculated. A two-sided *p*-value < 0.05 was considered statistically significant. Statistical analyses were performed using IBM SPSS Statistics version 31.0 (IBM Corp., Armonk, NY, USA).

## 3. Results

A total of 158 patients were included in the study. The median age at diagnosis was 63.5 years (IQR [57.00, 72.50]), and the median BMI was 29 kg/m^2^ (IQR [25.00, 34.00]).

The most frequent histological subtype was endometrioid carcinoma in 122 (77.2%) cases. Regarding molecular classification, 70 (44.3%) patients were NSMP, 46 (29.1%) were MMRd, 9 (5.7%) were POLE ultramutated, and 33 (20.9%) were p53-abn. No cases of multiple classification were found. Only one case shifted from NSMP to MMRd at the final pathological analysis. LVSI was present in 43 (27.2%) cases, and 128 (79.1%) patients had myometrial invasion (<50% in 51.3%, and ≥50% invasion in 27.8%).

According to the 2023 FIGO staging system, in the majority of cases, the tumor was confined to the uterus.

All patients underwent a primary surgical treatment consisting of total hysterectomy with bilateral salpingo-oophorectomy by a minimally invasive approach in 151 cases (93.8%).

Lymph node staging was performed in 151 (95.6%) patients, predominantly through SLNB in 147 patients (93%). In 7 patients, nodal staging was not performed due to surgical adhesions (n = 3), technical difficulties or comorbidities (n = 3), and intraoperative peritoneal carcinomatosis (n = 1). For descriptive analyses, these patients were retained in the cohort and classified according to the FIGO stage assigned based on the available surgical and pathological findings, despite incomplete nodal staging, in order to reflect routine clinical practice.

Adjuvant treatment was given to 82 (51.9%) patients.

When comparing the baseline, surgical, and pathological characteristics of the 158 patients depending on the molecular classification groups, differences in age at diagnoses (*p* = 0.026), FIGO stage at diagnoses (*p* = 0.001), ER (*p* = 0.002), PR (*p* < 0.001), and adjuvant therapy (*p* < 0.001) were found (Table 1).

Among 151 (95.6%) patients who underwent lymphatic assessment, SLNB was performed in 147 (93%) patients. Bilateral lymphadenectomy was completed in 14 (8.9%) patients.

A total of 18 (11.4%) patients had positive lymph nodes: 16 (10.1%) were SLNs, 1 (0.6%) was detected in pelvic lymphadenectomy, and 1 (0.6%) in paraaortic lymphadenectomy. Among the positive SLNs, 7 (4.4%) were micrometastasis, 8 (5.1%) macrometastasis, and 1 (0.6%) ITCs. Additionally, two macrometastases were detected in pelvic lymphadenectomy specimens (one in a patient with previously positive SLN), one micrometastasis in a pelvic lymph node (in a patient with positive SLN and radiologically suspicious lymphadenopathy), and one macrometastasis in a paraaortic lymphadenectomy specimen.

A comparative analysis was conducted across groups according to molecular subtypes to determine whether significant differences existed among them with respect to the number of positive lymph nodes. Differences were found in the rate of SLNB (*p* = 0.037) and the rates of total pelvic (*p* = 0.038) and paraaortic (*p* = 0.001) lymphadenectomy, with both greater in the p53-abn group. Moreover, significant differences in nodal involvement were observed across molecular subtypes (*p* = 0.010). No cases of nodal metastasis were detected in the POLE-mutated subgroup. The highest proportion of positive lymph nodes was observed in p53-abnormal tumors (24.2%), followed by NSMP (7.1%) and MMRd (6.5%) tumors. Regarding metastatic burden, macrometastases accounted for 50% of positive SLNs overall and were predominantly observed in the p53-abn subgroup, with a significant difference across molecular subgroups (*p* = 0.003). In contrast, all nodal metastases in the MMRd subgroup were micrometastases, while NSMP tumors showed a mixed pattern, including macrometastases, micrometastases, and ITCs. Only one case of ITCs was identified in the entire cohort, occurring in the NSMP subgroup. No differences were found for the other parameters studied, including the location of the sentinel lymph nodes. Table 2 shows a comparative analysis of lymph node assessment and lymph node status between molecular classification groups.

Regarding survival analysis, the median follow-up for the overall cohort was 27.5 months (IQR 16.0–37.5). According to the molecular subgroup, the median follow-up was 42.0 months (IQR 25.0–42.0) for POLE-mutated tumors, 27.5 months (IQR 16.0–36.0) for MMRd tumors, 21.0 months (IQR 14.0–32.0) for p53-abnormal tumors, and 30.0 months (IQR 18.3–41.8) for NSMP tumors, without statistically significant differences between groups (*p* = 0.075). At the last follow-up, 9 (5.7%) patients had died from the disease, and 22 (13.9%) patients experienced disease recurrence.

**Figure 1:**
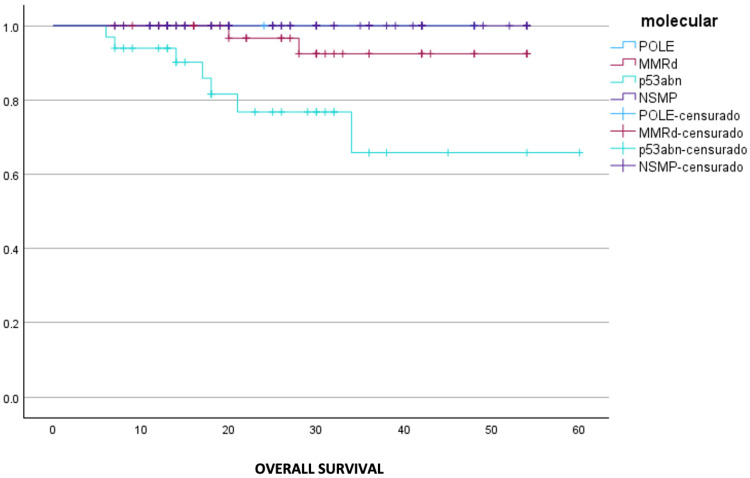
Overall survival comparing molecular classification groups (IBM SPSS Statistics 31.0 was used to create the figures).

Survival analysis according to molecular subtype revealed statistically significant differences in both OS and DFS. A Kaplan–Meier analysis demonstrated a significant association between molecular classification and OS (*p* < 0.001) (Figure 1). Patients classified as NSMP showed the most favorable outcomes, with the survival probabilities remaining above 90% throughout most of the follow-up period. The POLE-ultramutated group also exhibited a favorable prognosis, although the number of events was limited. The MMRd group displayed an intermediate prognosis, with a gradual and stepwise decline in survival, reaching approximately 75% toward the end of follow-up. In contrast, the p53-abn group was associated with the poorest OS, characterized by an earlier and more pronounced decline, with survival probabilities decreasing to around 60% after 30 months.

**Figure 2:**
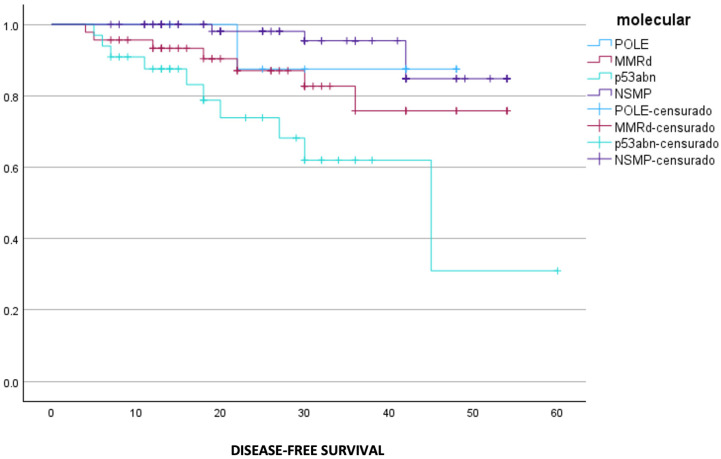
Disease-free survival comparing molecular classification groups. (IBM SPSS Statistics 31.0 was used to create the figures).

Differences in DFS were also statistically significant between groups (*p* < 0.01) (Figure 2). The NSMP and POLE-ultramutated groups maintained the highest DFS rates, remaining above 80% at the end of follow-up. The MMRd group showed a progressive reduction in DFS, crossing the 75% threshold at approximately 45 months. Remarkably, the p53-abn group demonstrated the poorest DFS, with earlier recurrences occurring before 10 months and a marked decline after 40 months, resulting in DFS rates close to 25% at the end of the study period.

Overall, on the univariate analysis evaluating prognostic factors for recurrence, high tumor grade (*p* = 0.024), LVSI (*p* = 0.012), deep myometrial invasion (*p* = 0.004), molecular classification (*p* = 0.02), and prognostic risk group according to FIGO 2023 (*p* = 0.038) were significantly associated with recurrence (Table 3).

Patients classified as intermediate risk and high risk according to FIGO 2023 had a significantly increased risk of recurrence compared with low-risk patients.

In the multivariate analysis, no variable retained independent statistical significance, although molecular classification and prognostic risk group showed a trend toward association with recurrence (Table 4).

Stratifying by molecular profile, in the univariate analysis, no statistically significant prognostic factors for disease recurrence were identified. In the MMRd subgroup, LVSI showed a strong trend toward increased recurrence risk (OR 5.17, 95% CI 0.96–27.91, *p* = 0.056). In the p53-abn group, a higher risk of recurrence was related to histology and LVSI, although this was not statistically significant. Overall, wide confidence intervals suggest limited precision, likely due to small sample size and a low number of events (Table 5).

In the multivariate analysis, no independent prognostic factors were identified. In the MMRd subgroup, LVSI remained associated with higher odds of recurrence (OR 6.20, 95% CI 0.66–59.13, *p* = 0.113), while lymph node involvement showed an increased risk without statistical significance (Table 6).

## 4. Discussion

In this cohort of surgically treated, early-stage endometrial cancer patients, molecular classification demonstrated significant associations with baseline clinicopathologic characteristics, nodal involvement patterns, and survival outcomes, reinforcing the central role in modern risk stratification, as initially described by TCGA [5], and subsequently validated in large translational series [18,19].

### 4.1. Baseline Characteristics and Molecular Distribution

The distribution of molecular subtypes in our cohort—NSMP as the most frequent, followed by MMRd, p53-abn, and POLE-ultramutated—is consistent with previously reported institutional and population-based datasets [18,19]. The significant differences observed in our cohort for age, FIGO stage, hormone receptor expression, and adjuvant therapy across molecular groups highlight the biological heterogeneity underlying endometrial carcinoma.

As expected, p53-abn tumors were more frequently associated with high-grade histology and advanced stage, consistent with their well-established aggressive phenotype [18,20]. In contrast, POLE-mutated tumors presented predominantly as uterine-confined disease with favorable characteristics, aligning with prior validation studies confirming their excellent prognosis, irrespective of grade [5,18].

The strong association between molecular classification and ER/PR expression in our series is particularly relevant in light of recent evidence demonstrating that hormone receptor status refines risk stratification, especially within NSMP tumors [21,22].

### 4.2. Nodal Assessment and Molecular Subtypes

Regarding lymph node assessment, SLNB was performed in the vast majority of cases, reflecting adherence to current ESGO and NCCN recommendations [2,10] and consistent with the diagnostic reliability established in prospective trials [11,23].

Importantly, nodal involvement differed significantly across molecular groups. The p53-abn subgroup exhibited a higher number of positive SLNs and greater paraaortic lymphadenectomy rates. Furthermore, macrometastases predominated within this group, supporting the hypothesis that molecular subtype may influence not only recurrence risk but also metastatic burden and dissemination pattern. In contrast, no nodal metastases were observed in POLE-mutated tumors, reinforcing the concept of biologically limited metastatic potential in this subgroup [5,18]. MMRd and NSMP tumors showed intermediate patterns of nodal involvement, consistent with their intermediate-risk classification in current molecular models.

Growing evidence suggests that molecular classification may contribute to predicting SLN involvement in endometrial cancer. However, its clinical utility remains debated. A meta-analysis by Luzarraga Aznar et al., including 3056 patients, reported higher rates of lymph node metastases in p53-abn and MMRd tumors (31% and 23%, respectively), whereas POLE-mutated tumors showed the lowest incidence [24]. Similarly, Cabrera et al. observed that p53-abn tumors were associated with higher rates of extrauterine disease and nodal involvement, supporting the integration of molecular profiling into preoperative risk assessment [25].

Other studies have confirmed this association. Praiss et al., in a cohort of 756 patients, demonstrated that p53-abn tumors were independently associated with an increased risk of micro- or macrometastasis [14]. Likewise, the SENECA study, a large retrospective analysis of 2139 patients with early-stage endometrial cancer, reported higher SLN involvement in p53-abn and MMRd subgroups compared with NSMP and POLE-mutated tumors [15]. Nevertheless, most SLN metastases corresponded to low-volume disease, raising questions about the clinical impact of these findings on treatment decisions.

Importantly, several studies suggest that conventional clinicopathologic features may still outperform molecular classification in predicting nodal involvement. The 2021 ESGO/ESTRO/ESP histopathologic risk stratification showed superior predictive accuracy for lymph node metastases, and results from the PROME trial indicated that factors such as LVSI and deep myometrial invasion remain stronger predictors of nodal disease than molecular features alone [26]. These findings highlight that, while molecular classification provides valuable biological insights, its independent predictive value for SLN metastases remains uncertain.

Overall, the current evidence supports a complementary rather than substitutive role of molecular profiling in SLN risk assessment. Integrating molecular data with established pathologic factors may improve risk stratification and guide treatment individualization. However, the retrospective design and heterogeneity of most available studies limit definitive conclusions. Prospective studies, including the ongoing EUGENIE trial, are needed to clarify the clinical relevance of molecular classification in predicting SLN involvement and guiding management in early-stage endometrial cancer [27].

These findings suggest that molecular classification may inform surgical decision-making beyond traditional histopathologic parameters, potentially guiding the intensity of nodal evaluation and adjuvant therapy selection.

### 4.3. Survival Outcomes According to Molecular Subtype

The survival analysis demonstrated statistically significant differences in both OS and DFS across molecular subtypes. Patients with p53-abn tumors exhibited the poorest outcomes, with early recurrences and marked decline in survival probabilities, confirming prior PORTEC-3 and TransPORTEC analyses that demonstrated inferior outcomes in this subgroup despite adjuvant therapy [18,20]. POLE-ultramutated patients maintained excellent survival throughout follow-up, consistent with their ultramutated, immune-activated phenotype and favorable clinical behavior [5,18]. The MMRd group showed intermediate survival outcomes. Although MMR deficiency confers sensitivity to immune checkpoint inhibitors in advanced and recurrent settings—as shown in KEYNOTE-775—its prognostic role in early-stage disease remains intermediate rather than clearly favorable [28].

The NSMP group demonstrated favorable survival in our cohort. However, recent studies have demonstrated that ER expression significantly refines prognosis within NSMP tumors [21,22,29,30]. ER-negative NSMP cases behave more aggressively, approaching outcomes seen in p53-abn disease, whereas ER-positive NSMP tumors show excellent survival and may benefit from hormonal therapy strategies. The high prevalence of ER positivity in our NSMP cohort likely contributed to the favorable outcomes observed. The 2025 ESGO update further supports the integration of hormone receptor status into molecular risk stratification [2], reinforcing the concept that NSMP should not be considered a single prognostic entity.

### 4.4. Prognostic Factors for Recurrence

In the overall cohort, univariate analysis identified histologic grade, LVSI, deep myometrial invasion, FIGO stage, and molecular classification as significantly associated with recurrence. These findings are consistent with established prognostic frameworks and the 2023 FIGO staging revision [31], reinforcing the continued relevance of traditional clinicopathologic factors in risk assessment. However, none of these variables retained independent statistical significance in multivariate analysis, probably due to the relatively limited number of recurrence events.

Importantly, our results are consistent with the analysis of the GOG-0258 trial [32], which demonstrated that molecular classification provides independent prognostic information for both DFS and OS. In that study, p53-abn tumors were associated with the highest risk of recurrence and poorest survival outcomes, whereas POLE-mutated tumors showed excellent prognosis, regardless of adverse clinicopathologic features. MMRd and NSMP tumors displayed intermediate outcomes, mirroring the survival patterns observed in our cohort. These findings support the hypothesis that molecular classification refines and, in some contexts, may replace traditional prognostic factors.

When analyzing the prognostic factors stratified by molecular subtype (Table 5 and Table 6), distinct patterns emerged that further highlight the biological heterogeneity of endometrial cancer. In the POLE-mutated subgroup, no variables were associated with recurrence, supporting the well-established concept that these tumors have an intrinsically favorable prognosis independent of histologic or pathologic features [15].

In the MMRd subgroup, LVSI demonstrated a strong trend toward increased recurrence risk in both univariate (OR 5.17, *p* = 0.056) and multivariate analyses (OR 6.20), suggesting that LVSI may represent a key driver of recurrence within this molecular category. Additionally, lymph node involvement showed a consistent, although non-significant, association with recurrence risk, indicating that metastatic dissemination may still play a relevant role in this subgroup. These findings are clinically meaningful, as they suggest that, despite the intermediate prognosis typically attributed to MMRd tumors, the presence of LVSI could identify a higher-risk subset that may benefit from treatment intensification.

In contrast, in the p53-abn subgroup, histology and LVSI were associated with an increased risk of recurrence in a univariate analysis, although without reaching statistical significance. This likely reflects the intrinsically aggressive biology of this subtype, in which recurrence risk is high regardless of additional clinicopathologic modifiers. This observation is consistent with GOG-0258 data, where p53-abn tumors exhibited poor outcomes independent of other risk factors, suggesting that molecular subtype itself may be the dominant prognostic determinant in this group [32].

Within the NSMP subgroup, conventional factors such as grade and LVSI showed trends toward increased recurrence risk, although with no statistical significance. This heterogeneity aligns with emerging evidence indicating that NSMP tumors represent a biologically diverse group, in which additional biomarkers—particularly hormone receptor status—are required to refine prognostic stratification [2]. The high prevalence of ER positivity in our cohort may explain the relatively favorable outcomes observed, masking potential risk differences within this subgroup.

Overall, the absence of statistically significant independent predictors in multivariate analyses across molecular subtypes is likely influenced by limited sample size and event rates. Nevertheless, the consistent trends observed—particularly the impact of LVSI in MMRd tumors and the dominant effect of molecular subtype in p53-abn disease—support the concept that prognostic factors operate differently across molecular categories.

All these findings reinforce the need for an integrated prognostic model that combines molecular classification with selected clinicopathologic variables. Rather than applying uniform risk factors across all patients, subtype-specific risk stratification may provide a more accurate prediction of recurrence and guide individualized therapeutic strategies [32].

### 4.5. Clinical Implications and Future Directions

By combining molecular profiling with SLN mapping in a contemporary surgical cohort, our study bridges biological risk stratification and real-world surgical staging. The differential nodal burden observed across molecular subtypes—particularly in p53-abn tumors—highlights the potential of integrating molecular data into surgical and adjuvant decision-making algorithms.

The strengths of this study include adherence to ESGO guidelines [4], WHO-based molecular classification [5], standardized SLN ultrastaging, and comprehensive survival analysis. Limitations include its retrospective design, limited follow-up duration (particularly given the inclusion period from 2021 to 2024, which may limit the detection of late recurrences and affect the robustness of survival estimates), and the limited sample size in specific molecular subgroups, particularly the small number of POLE-mutated cases. The retrospective nature of the analysis may introduce inherent selection and information biases, as well as heterogeneity in clinical management and follow-up practices over time. In addition, although the cohort is well-characterized, the overall sample size, and especially the low number of events within certain subgroups, reduces the statistical power of the study. This is particularly relevant for the POLE-mutated subgroup and for stratified and multivariable analyses, where wide confidence intervals were observed, and some associations did not reach statistical significance. Moreover, the limited number of recurrence events relative to the number of variables included in the multivariable models raises the possibility of model overfitting, and alternative approaches such as penalized regression may be more appropriate in this setting. Furthermore, this is a single-institution study without an independent validation cohort, which may limit the generalizability of the findings. Although our results are consistent with previously published evidence, they should be interpreted with caution and considered hypothesis-generating. External validation in larger, independent, and preferably multicenter cohorts is warranted to confirm the reproducibility and broader applicability of these findings. In addition, detailed information on the specific types of adjuvant therapies administered (including radiotherapy, chemotherapy, chemoradiotherapy, immunotherapy, or hormonal therapy) was not systematically available for all patients and, therefore, could not be analyzed in relation to molecular subgroups. This limitation prevents a more detailed evaluation of how differences in treatment may have influenced survival outcomes and constitutes an important source of potential residual confounding.

Despite these limitations, our findings support a transition from morphology-based staging toward an integrated biologically driven model that incorporates molecular subtype, hormone receptor status, and SLN assessment.

By aligning molecular classification, hormonal biomarkers, and nodal staging within a single institutional cohort, this study contributes clinically applicable evidence supporting precision oncology approaches in endometrial cancer—an area of growing relevance in contemporary translational research and guideline development.

## 5. Conclusions

Molecular classification demonstrated significant associations with nodal involvement patterns and survival outcomes in this cohort of early-stage endometrial cancer patients. The p53-abn subgroup showed the highest nodal metastatic burden and the poorest survival, whereas POLE-mutated tumors exhibited excellent prognosis with no nodal involvement, confirming their intrinsically favorable biological behavior. MMRd and NSMP tumors presented intermediate outcomes, highlighting the heterogeneity within these categories.

Although traditional clinicopathologic variables such as grade, LVSI, and myometrial invasion remained associated with recurrence risk in the univariate analysis, their prognostic impact appeared to vary according to molecular subtype. In particular, LVSI showed a consistent trend toward increased recurrence risk in MMRd tumors, suggesting that the prognostic factors may operate differently across molecular categories.

## Figures and Tables

**Table 1:** Baseline, surgical, and pathological characteristics from total cohort and molecular classification groups. Data are given in median [interquartile range] or frequencies (percentages).

	Overall (n = 158)	POLE-Mut (n = 9)	MMRd (n = 46)	p53-abn (n = 33)	NSMP (n = 70)	*p* Value
**Age at diagnoses (years)**	63.50 [57.00, 72.50]	57.00 [53.00, 61.00]	65.00 [51.75, 74.50]	67.00 [63.00, 74.00]	61.00 [56.25, 68.75]	**0.026**
**BMI (kg/m^2^)**	29.00 [25.00, 34.00]	27.00 [25.00, 30.00]	29.00 [25.00, 35.00]	29.00 [25.00, 33.00]	30.00 [25.00, 35.75]	0.624
**FIGO 2023 stage**						**0.001**
I	100 (63)	8 (9)	33 (71.7)	6 (18.1)	53 (75.7)	
II	32 (20)	0 (0.0)	9 (19.5)	14 (42.4)	9 (12.8)	
III	24 (15)	1 (11.1)	4 (8.7)	11 (33.3)	8 (11.4)	
IV	2 (1)	0 (0.0)	0 (0.0)	2 (6)	0 (0.0)	
**Surgical approach**						0.613
Laparoscopic	133 (84.2)	8 (88.9)	36 (78.3)	30 (90.9)	59 (84.3)	
Robotic	18 (11.4)	1 (11.1)	8 (17.4)	1 (3.0)	8 (11.4)	
Open	7 (4.4)	0 (0.0)	2 (4.3)	2 (6.1)	3 (4.3)	
**Lymph node assessment**						0.253
No	7 (4.4)	0 (0.0)	3 (6.5)	3 (9.1)	1 (1.4)	
Yes	151 (95.6)	9 (100)	43 (93.5)	30 (90.9)	69 (98.6)	
**Histological subtype**						<0.001
Low-grade endometrioid	111 (70.2)	6 (66.7)	38 (82.6)	2 (6.1)	65 (92.9)	
High-grade endometrioid	13 (8.2)	2 (22.2)	4 (8.7)	3 (9.1)	4 (5.7)	
Serous carcinoma	20 (12.6)	1 (11.1)	1 (2.2)	18 (54.5)	0 (0.0)	
Clear cell carcinoma	3 (1.9)	0 (0.0)	0 (0.0)	3 (9.1)	0 (0.0)	
Undifferentiated	5 (3.2)	0 (0.0)	2 (4.3)	2 (6.1)	1 (1.4)	
Carcinosarcoma	5 (3.2)	0 (0.0)	0 (0.0)	5 (15.1)	0 (0.0)	
Others	1 (0.6)	0 (0.0)	1 (2.2)	0 (0.0)	0 (0.0)	
**LVSI**						0.212
Positive	43 (27.2)	5 (55.6)	12 (26.1)	10 (30.3)	16 (22.9)	
Negative	115 (72.8)	4 (44.4)	34 (73.9)	23 (69.7)	54 (77.1)	
**Myometrial invasion**						0.057
Absence	33 (20.9)	0 (0.0)	11 (23.9)	9 (27.3)	13 (18.6)	
<50%	81 (51.3)	9 (100.0)	24 (52.2)	12 (36.4)	36 (51.4)	
≥50%	44 (27.8)	0 (0.0)	11 (23.9)	12 (36.4)	21 (30.0)	
ER *						**0.002**
Negative	6 (3.8)	0 (0.0)	0 (0.0)	5 (15.2)	1 (1.4)	
Positive	150 (96.2)	9 (100.0)	44 (100.0)	28 (84.8)	69 (98.6)	
PR **						**<0.001**
Negative	15 (9.8)	0 (0.0)	3 (7.0)	10 (32.3)	2 (2.9)	
Positive	138 (90.2)	9 (100.0)	40 (93.0)	21 (67.7)	68 (97.1)	
**Adjuvant therapy**						**<0.001**
No	72 (45.6)	6 (66.7)	24 (52.2)	3 (9.1)	39 (55.7)	
Yes	82 (51.9)	2 (22.2)	21 (45.7)	29 (87.9)	30 (42.9)	
**Prognostic classification**						
Low risk	76 (48.1)	8 (88.9)	27 (58.7)	0 (0)	41 (58.6)	**<0.001**
Intermediate risk	21 (13.3)	0 (0)	9 (19.6)	0 (0)	12 (17.1)	
Intermediate–high risk	10 (6.3)	0 (0)	4 (8.7)	0 (0)	6 (8.6)	
High risk	39 (24.7)	0 (0)	4 (8.7)	27 (81.8)	8 (11.4)	
Indeterminate	12 (7.6)	1 (11.1)	2 (4.3)	6 (18.2)	3 (4.3)	

BMI: body mass index; LVSI: lymph vascular space invasion; ER: estrogen receptor; PR: progesterone receptor. * missing data: 2 patients; ** missing data: 5 patients. Statistically significant variables are highlighted in bold.

**Table 2:** Comparative analysis of lymph node assessment and lymph node status between molecular classification groups. Data are given in mean [standard deviation] or frequencies (percentages).

	Overall (n = 158)	POLE-Mut (n = 9)	MMRd (n = 46)	p53-abn (n = 33)	NSMP (n = 70)	*p* Value
**Lymph node assessment**						
No	7 (4.4)	0 (0.0)	3 (6.5)	3 (9.1)	1 (1.4)	0.253
Yes	151 (95.6)	9 (100)	43 (93.5)	30 (90.9)	69 (98.6)	
**SNLB**						**0.037**
*No*	11 (7)	0 (0.0)	5 (10.9)	5 (15.2)	1 (1.4)	
*Yes*	147 (93.0)	9 (100.0)	41 (89.1)	28 (84.8)	69 (98.6)	
**Pelvic lymphadenectomy**						
No	144 (91.1)	8 (88.9)	43 (93.5)	26 (78.8)	67 (95.7)	**0.038**
Yes	14 (8.9)	1 (11.1)	3 (6.5)	7 (21.2)	3 (4.3)	
**Paraaortic lymphadenectomy**						
No	150 (94.9)	8 (88.9)	45 (97.8)	27 (81.8)	70 (100.0)	**0.001**
Yes	8 (5.1)	1 (11.1)	1 (2.2)	6 (18.2)	0 (0.0)	
**Number of SLNs removed**	2.32 [±1.029]	3.0 [±1.323]	2.2 [±0.98]	2.06 [±1.144]	2.43 [±0.926]	0.057
**Number of positive SLNs**	16 (10.1)	0 (0)	3 (6.5)	8 (24.2)	5 (7.1)	**0.010**
Macrometastasis	8 (50)	0 (0)	0 (0)	7 (87.5)	1 (20)	**0.003**
Micrometastasis	7 (43.8)	0 (0)	3 (100)	1 (12.5)	3 (60)	
ITCs	1 (6.2)	0 (0)	0 (0)	0 (0)	1 (20)	
**Number of pelvic LNs removed**	0.77 [±2.661]	2.0 [±4.69]	0.65 [±2.61]	1.61 [±3.774]	0.30 [±1.323]	0.057
**Number of positive pelvic LNs**	0.02 [±0.177]	0	0	0.06 [±0.348]	0.01 [±0.120]	0.480
**Number of paraaortic LNs removed**	0.56 [±2.422]	1.56 [±3.127]	0.35 [±2.359]	1.76 [±4.00]	0	**0.003**
**Number of positive paraaortic LNs**	0.03 [±0.224]	0	0	0.12 [±0.485]	0	0.053
**Location of right SLNs**						0.227
Right SLN	143 (90.5)	9 (100)	40 (86.9)	27 (81.8)	67 (95.7)	
Obturator	97 (67.8)	6 (66.7)	31 (77.5)	15 (55.6)	45 (67.2)	
External iliac	42 (29.4)	3 (33.3)	7 (17.5)	10 (37)	22 (32.8)	
Presacral	2 (1.4)	0 (0)	1 (2.5)	1 (3.7)	0 (0)	
Common iliac	2 (1.4)	0 (0)	1 (2.5)	1 (3.7)	0 (0)	
**Location of left SLNs**						0.089
Left SLN	141 (89.2)	9 (100)	41 (89.1)	25 (75.8)	66 (94.3)	
Obturator	73 (51.8)	6 (66.7)	23 (56.1)	11 (44)	33 (50)	
External iliac	68 (48.2)	3 (33.3)	18 (43.9)	14 (56)	33 (50)	

MMRd: mismatch repair deficiency; abn: abnormal; NSMP: no specific molecular profile; SLNB: sentinel lymph node biopsy; LNs: lymph nodes; SLNs: sentinel lymph nodes; ITCs: isolated tumor cells. Statistically significant variables are highlighted in bold.

**Table 3:** Prognostic factors associated with disease recurrence, overall (univariate analysis).

Variable	OR	CI 95%	*p* Value
**Histology**	2.42	0.92–6.37	0.074
**Grade**	2.89	1.15–7.24	**0.024**
**LVSI**	3.25	1.29–8.2	**0.012**
**Myometrial invasion**	3.9	1.54–9.87	**0.004**
**Lymph node involvement**	2.78	0.88–8.78	0.081
**Molecular classification**			**0.02**
MMRd vs. POLEmut	1.44	0.16–13.34	0.75
p53-abn vs. POLEmut	3.48	0.38–31.63	0.268
NSMP vs. POLEmut	0.49	0.05–4.89	0.539
**Prognostic classification (FIGO 2023)**			**0.038**
Intermediate vs. low risk	7.6	1.65–35.12	**0.009**
Intermediate–high vs. low risk	6.08	0.88–42.01	0.067
High vs. low risk	8.39	2.15–32.69	**0.002**
Indeterminate vs. low risk	4.87	0.72–32.78	0.104

LVSI: lymph vascular space invasion; MMRd: mismatch repair deficiency; p53-abn: p53 abnormal; NSMP: no specific molecular profile; POLE mut: POLE mutated; OR: odds ratio; CI: confidence interval. Statistically significant variables are highlighted in bold.

**Table 4:** Prognostic factors associated with disease recurrence overall (multivariate analysis).

Variable	OR	CI 95%	*p* Value
Grade	0.542	0.12–2.41	0.420
Lymphovascular space invasion	2.08	0.64–6.82	0.225
Myometrial invasion	3.47	0.62–9.98	0.201
Molecular classification			0.108
Prognostic classification			0.583

OR: odds ratio; CI: confidence interval.

**Table 5:** Prognostic factors associated with disease recurrence by molecular profile (Univariate analysis).

Molecular Subtype	Variable	OR	95% CI	*p* Value
POLEmut	Histology	0.87	0.67–1.13	0.889
	LVSI	0.75	0.43–1.32	0.444
MMRd	Histology	1.16	0.66–2.03	0.600
	LVSI	5.17	0.96–27.91	0.056
	Grade	0.90	0.11–7.03	0.920
	Myometrial invasion	0.38	0.06–2.29	0.292
	Lymph node involvement	3.08	0.24–39.51	0.387
p53-abn	Histology	4.50	0.62–32.69	0.137
	LVSI	3.60	0.74–17.59	0.114
	Grade	1.35	0.12–14.82	0.806
	Myometrial invasion	2.67	0.23–31.07	0.434
	Lymph node involvement	2.40	0.48–11.97	0.286
NSMP	Histology	0.94	0.88–0.99	0.943
	LVSI	3.71	0.48–28.76	0.209
	Grade	3.69	0.47–28.78	0.212

LVSI: lymphovascular space invasion; MMRd: mismatch repair deficiency; p53-abn: p53 abnormal; NSMP: no specific molecular profile; POLE mut: POLE mutated; OR: odds ratio; CI: confidence interval.

**Table 6:** Prognostic factors associated with disease recurrence by molecular profile (multivariate analysis).

Molecular Subtype	Variable	OR	95% CI	*p* Value
MMRd	Histology	1.11	0.47–2.59	0.819
	LVSI	6.20	0.66–59.13	0.113
	Grade	2.24	0.07–70.09	0.646
	Myometrial invasion	1.26	0.05–32.93	0.888
	Lymph node involvement	2.31	0.08–64.01	0.621
p53-abn	Histology	1.04	0.57–1.78	0.988
	LVSI	1.92	0.29–12.77	0.500
	Grade	2.27	0.14–37.97	0.568
	Myometrial invasion	2.16	0.16–28.98	0.558
	Lymph node involvement	1.34	0.21–8.79	0.759
NSMP	LVSI	1.52	0.13–18.59	0.741
	Grade	1.52	0.13–18.59	0.741

LVSI: lymphovascular space invasion; MMRd: mismatch repair deficiency; p53-abn: p53 abnormal; NSMP: no specific molecular profile; OR: odds ratio; CI: confidence interval.

## Data Availability

The data presented in this study are available on request from the corresponding author.

## Abbreviations

The following abbreviations are used in this manuscript:

EC	Endometrial cancer
WHO	World Health Organization
POLE	DNA polymerase epsilon
MMR	Mismatch repair
MMRd	Mismatch repair deficient
p53-abn	p53 abnormal
NSMP	No specific molecular profile
ER	Estrogen receptor
PR	Progesterone receptor
TCGA	The Cancer Genome Atlas
ESGO	European Society of Gynaecological Oncology
PFS	Progression-free survival
OS	Overall survival
SLN	Sentinel lymph node
SLNB	Sentinel lymph node biopsy
NCCN	National Comprehensive Cancer Network
LVSI	Lymphovascular space invasion
MRI	Magnetic resonance imaging
CT	Computed tomography
PET-CT	Positron emission tomography–computed tomography
MIS	Minimally invasive surgery
ICG	Indocyanine green
ITCs	Isolated tumor cells
BMI	Body mass index
FIGO	International Federation of Gynecology and Obstetrics
IQR	Interquartile range
SD	Standard deviation
OR	Odds ratio
CI	Confidence interval
MSI-H	Microsatellite instability—high
RFS	Relapse-free survival
